# Social Engagement and Episodic Memory in Non-Hispanic Black and White Older Adults

**DOI:** 10.1093/geroni/igaa057.1101

**Published:** 2020-12-16

**Authors:** Abbey Hamlin, A Zarina Kraal, Laura Zahodne

**Affiliations:** 1 University of Michigan, Highland, Michigan, United States; 2 University of Michigan, Ann Arbor, Michigan, United States

## Abstract

Social engagement may confer cognitive benefits in older adulthood, but studies have typically been restricted to largely non-Hispanic White (NHW) samples. Levels of social engagement vary across race such that NHW report larger social networks, more frequent participation in social activities, and greater social support than non-Hispanic Blacks (NHB). Associations between social engagement and cognition may also vary by race, but research is sparse. The current cross-sectional study examined associations between different aspects of social engagement and episodic memory performance, as well as interactions between social engagement and race among NHB and NHW participants in the Michigan Cognitive Aging Project (N = 247; 48.4% NHB; age = 64.19 ± 2.92). Social engagement (network size, activities, support) was self-reported. Episodic memory was a z-score composite of immediate, delayed, and recognition trials of a list-learning task. Separate hierarchical linear regression models quantified interactions between race and each of the three social engagement variables on episodic memory, controlling for sociodemographics, depressive symptoms, and health conditions. Results showed a main effect of more frequent social activity on better episodic memory, as well as an interaction between race and social support indicating a significant positive association in NHB but not NHW. These preliminary findings suggest that participating in social activities may be equally beneficial for episodic memory across NHB and NHW older adults and that social support may be particularly beneficial for NHB. Future research is needed to determine the potential applications of these results in reducing cognitive inequalities through the development of culturally-relevant interventions.

